# Factors influencing breast and cervical cancer screening among ever-married women aged 15–49 in Jordan: an analysis of the 2023 Jordan population and family health survey

**DOI:** 10.1186/s43046-025-00320-z

**Published:** 2025-09-26

**Authors:** Rajat Das Gupta, Shuvajit Saha, Md Ataur Rahman, Prince NII Ossah Addo, Rohan Kothadia, Georgios Vasilios Lahanas, Ananna Mazumder, Arpan Das Gupta, Ehsanul Hoque Apu, Nazeeba Siddika

**Affiliations:** 1 Department of Epidemiology and Biostatistics, University of South Carolina, Columbia, United States; 2 Centre for International Public Health and Environmental Research, Dhaka, Bangladesh; 3 Usher Institute, University of Edinburgh, Edinburgh, United Kingdom; 4 Department of Oncology, Karmanos Cancer Institute, Detroit, United States; 5 New York University College of Dentistry, New York City, United States; 6 Undergraduate Academic Program, Arnold School of Public Health, University of South Carolina, Columbia, United States; 7 Department of Public Health, School of Health and Life Science, North South University, Dhaka, Bangladesh; 8 Department of Development Studies, Daffodil International University, Dhaka, Bangladesh; 9 Department of Biomedical Sciences, Northeast Ohio Medical University, 4209 State Route 44, Rootstown, OH United States; 10 Department of Specialty Dentistry, Bitonte College of Dentistry, Northeast Ohio Medical University, 4209 State Route 44, Rootstown, OH United States; 11 Center for Global Health Equity, University of Michigan–Ann Arbor, Michigan, United States

**Keywords:** Breast cancer, Cervical cancer, Early detection of cancer, Mass screening

## Abstract

**Purpose:**

This study sought to investigate the prevalence and sociodemographic determinants related to breast and cervical cancer screening among ever-married women aged 15 to 49 years in Jordan.

**Methods:**

This research employed secondary data from the 2023 Jordan Population and Family Health Survey (JPFHS), which included 12,547 ever-married women aged 15 to 49. Weighted multivariable logistic regression analyses were conducted to quantify screening prevalence and identify related covariates, presented as adjusted odds ratios (AORs) with 95% confidence intervals (CIs).

**Results:**

The prevalence of screening for breast and cervical cancer was 15.2% and 16.2%, respectively. Increased screening participation was substantially correlated with advanced age, larger home affluence, higher parity, previous sexually transmitted infections (STIs), and exposure to radio communications. Women aged 35–49 were more likely to receive breast (AOR: 4.0; 95% CI: 2.6–6.0) and cervical cancer screening (AOR: 5.5; 95% CI: 3.3–9.2) compared to those aged 15–24 years. Women in the highest wealth quintile had a greater likelihood of being screened for breast cancer (AOR: 2.1; 95% CI: 1.6–2.8) and cervical cancer (AOR: 2.6; 95% CI: 1.9–3.5). Moreover, breast cancer screening correlated with recent healthcare service consumption (AOR: 1.3; 95% CI: 1.1–1.6), while cervical cancer screening had a favorable association with elevated educational attainment (AOR: 1.6; 95% CI: 1.2–2.3). Living in rural areas was inversely correlated with cervical screening participation (AOR: 0.7; 95% CI: 0.6–1.0).

**Conclusion:**

Screening rates for breast and cervical cancer among Jordanian women are inadequate. Interventions that facilitate equitable access—especially aimed at younger, less educated, rural, and low-income women—are crucial for enhancing participation and diminishing inequities in early cancer detection.

## Introduction

Breast and cervical cancers are among the leading causes of morbidity and mortality among women worldwide, with a particularly high burden observed in low- and middle-income countries (LMICs) [1–4]. In the Middle East and North Africa (MENA) region, breast cancer accounted for approximately 25% of all cancer cases and nearly 20% of cancer-related deaths among women in 2020, while cervical cancer contributed an estimated 89,800 new cases and over 47,500 deaths [3, 4]. Jordan, a MENA country with a population of approximately 10.1 million, 48.5% of whom are women, mirrors these regional trends [5–7]. In Jordan, breast cancer accounted for 20.1% of all reported cancer cases in 2022, with 1756 new cases among women, while cervical cancer had a lower crude incidence rate of 2.3 per 100,000 women, with 277 new cases in 2020, though this may underestimate the true burden due to limitations in cancer registration and reporting [6, 7]. 

Screening methods such as mammography for breast cancer and Pap smear for cervical cancer can identify abnormalities before symptoms appear, allowing for earlier and more effective treatment [8, 9]. Early-stage cancers are typically more treatable and associated with better survival outcomes. In low-resource settings, where late-stage presentation is common due to limited access to healthcare, implementing and scaling up screening programs is vital for reducing the disease burden [10]. In low- and middle-income countries (LMICs), cervical cancer is often detected at a late stage, primarily due to the lack of systematic screening and early detection programs. Approximately 80% of cancer patients in these settings seek health care only after the disease has progressed to an advanced stage, limiting treatment success [11]. Cervical cancer mortality remains disproportionately high in LMICs, accounting for more than 85% of global deaths, largely due to gaps in screening and timely treatment [12]. Similarly, breast cancer is frequently diagnosed at advanced stages in LMICs. In Egypt, for example, pooled estimates indicate that 56% of women are diagnosed at advanced stages, with comparable patterns observed across Latin America and the Caribbean [13]. Contributing factors include inadequate screening coverage, limited public awareness, and initial reliance on traditional health practices before engaging with formal healthcare systems [13]. Evidence from a systematic review and meta-analysis commissioned by the U.S. Preventive Services Task Force demonstrates that mammography screening modestly reduces breast cancer mortality, particularly in women aged 50 to 69 years, with relative risks (RR) ranging from 0.67 to 0.86. The greatest benefit was observed among women aged 60 to 69 years, corresponding to 21 deaths averted per 10,000 women screened over a 10-year period. Screening also reduced the incidence of advanced breast cancer among women aged 50 years and older (RR = 0.62), although no significant reduction in all-cause mortality was observed. The evidence was limited for women under 50 or over 70 years, and most included trials employed outdated imaging and treatment protocols [14]. A separate systematic review conducted for the Canadian Task Force on Preventive Health Care reported that cervical cancer screening substantially reduces both mortality and incidence. A randomized controlled trial in India showed that even a single lifetime screening could reduce cervical cancer mortality (RR = 0.65) and incidence of advanced disease (RR = 0.56). Supporting evidence from cohort and case–control studies indicated a 65% reduction in cervical cancer risk associated with cytology screening [15, 15]. However, despite national initiatives such as the Jordan Breast Cancer Program (JBCP), which screened over 6000 women in 2024 through mobile units and partnerships with private hospitals, participation in breast cancer screening programs remains suboptimal [16].

According to data from the 2018 Jordan Population and Family Health Survey (JPFHS 2018), breast cancer screening rates among ever-married women aged 15 to 49 remain notably low. Only 13.9% of women in this group reported having undergone a breast examination by a specialist within the previous year. Furthermore, just 8.7% received a mammogram. Awareness of cervical cancer screening is somewhat more widespread, with approximately 65% of ever-married women in the same age range indicating familiarity with the Pap smear test. However, among those who are aware of the test, only 24% have undergone the procedure. Patterns in the data suggest that knowledge of and participation in Pap testing tend to improve with higher age and educational attainment. Additionally, wealthier women were more likely to have heard of and received the test, reflecting a positive correlation between socioeconomic status and access to or utilization of screening services [17]. Similarly, a recent study in Jordan identified several factors influencing participation in breast cancer screening services, including limited access to information and resources, physical accessibility of screening facilities, financial constraints, religious and cultural beliefs, lack of social support, and prevailing societal norms [6]. These barriers can lead to delayed diagnosis and treatment, often resulting in advanced-stage presentation and poorer survival outcomes. Addressing these challenges is therefore critical to improving early detection and reducing the burden of breast cancer in the region.

Despite previous studies [17–20], a significant gap remains in the literature regarding a comprehensive, nationally representative analysis of the determinants influencing participation in breast and cervical cancer screening in Jordan using the most recent data. Previous research studies have either been constrained in scope or concentrated on specific subpopulations, restricting their results’ generalizability. Given the increasing cancer burden and consistently low screening participation rates, it is essential to investigate the sociodemographic and environmental factors that influence screening habits nationally. This study fills this gap by employing data from the 2023 Jordan Population and Family Health Survey (JPFHS 2023), providing substantial evidence to direct focused public health activities and shape national cancer control policies.

## Methods

### Study design

This study is based on secondary data from the JPFHS 2023, the eighth such survey conducted in Jordan. The JPFHS 2023 was implemented by the Department of Statistics (DoS) at the request of the Ministry of Health, with data collection carried out between January 2 and June 15, 2023. The primary aim of the survey was to generate nationally representative and up-to-date estimates of key demographic and health indicators to inform evidence-based health planning and policy [21].

The JPFHS was supported technically by ICF through The DHS Program and financially by the United States Agency for International Development (USAID), along with contributions from the Government of Jordan, UNICEF, UNFPA, WHO, and WFP. Key objectives included assessment of fertility trends, childhood mortality, contraceptive use, maternal and child health, nutritional status, anemia prevalence, HIV/AIDS awareness, experience of gender-based violence, and disability. The findings are intended to support policymakers and program managers in designing and evaluating strategies aligned with national health priorities and the Sustainable Development Goals (SDGs) for Jordan. The final report of JPFHS 2023, including survey methods and results, was published previously [21].

**Fig. 1:**
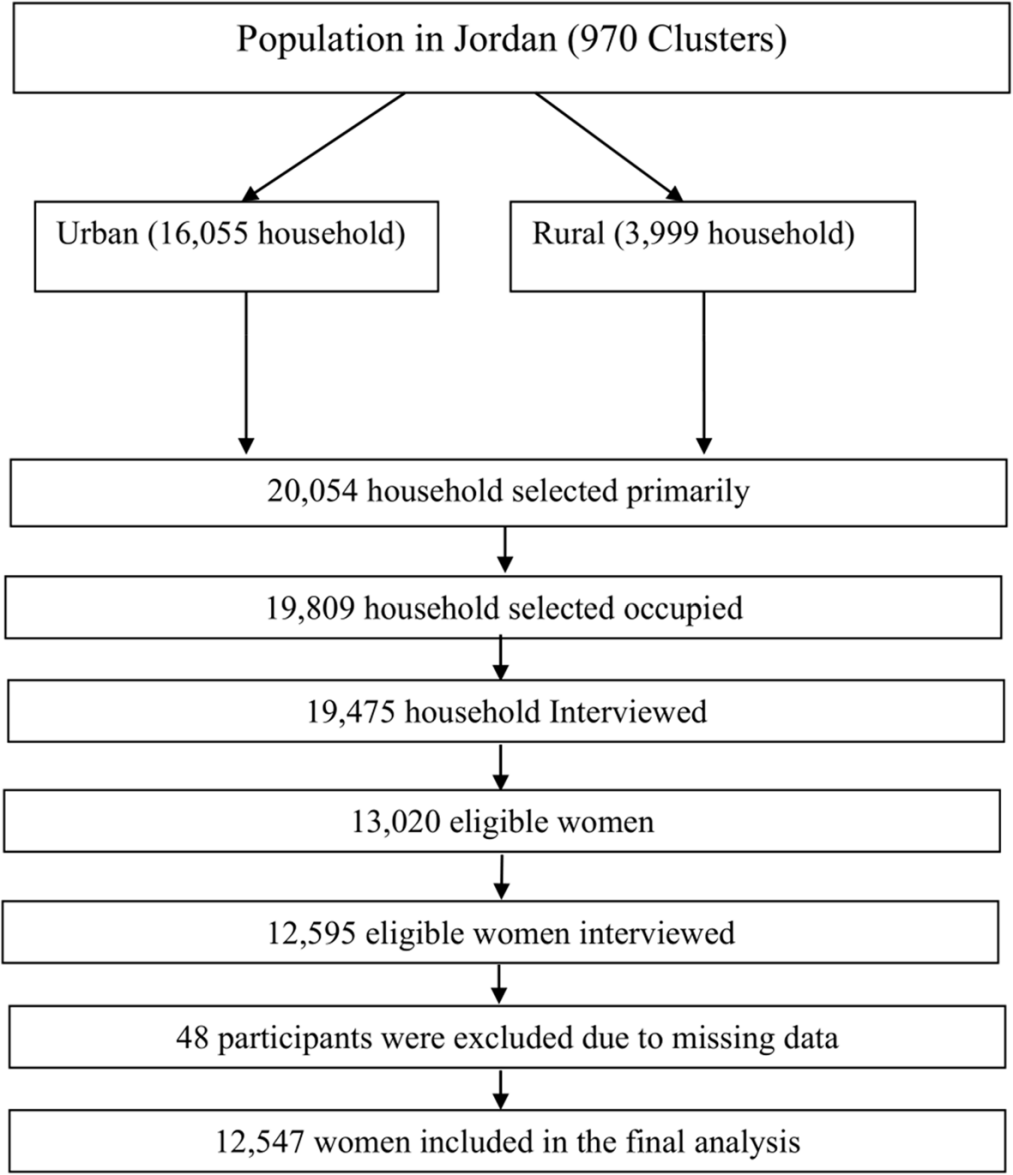
Flowchart showing the process of selecting the participants in the survey

The JPFHS 2023 used a two-stage stratified cluster sampling design based on the 2015 Jordan Population and Housing Census. The sampling frame was constructed using census blocks grouped into clusters, which served as the primary sampling units. A total of 970 clusters were selected with probability proportional to size, stratified by urban/rural residence across the 12 governorates, with additional strata for Syrian refugee camps in Zarqa and Mafraq, yielding 26 total strata. Within each selected cluster, 20 households were systematically sampled. Eligible respondents included ever-married women aged 15–49 who were usual residents or present the night before the survey [21]. The study’s analytic sample selection process is shown in Fig. 1.

### Data collection instruments, techniques, and collection

The JPFHS 2023 used five questionnaires for data collection: the household questionnaire, the woman’s questionnaire, the man’s questionnaire, the biomarker questionnaire, and the fieldworker questionnaire. Data for this study were drawn from the household and women’s questionnaires. Information on women’s demographic and health characteristics was obtained from the women’s questionnaire, while household-level data used to construct the wealth index came from the household questionnaire. These pretested questionnaires, adapted from the DHS Program’s models, addressed population and health issues specific to Jordan [21]. This study did not include information from the man’s questionnaire, the biomarker questionnaire, and the fieldworker questionnaire.

Pretest training was held in Amman from October 2 to 17, 2022, followed by field testing in selected clusters from October 18 to 20. Based on the pretest, adjustments were made to the questionnaires. Main field staff training occurred from November 20 to December 24, 2022, involving 200 staff members, with additional biomarker training provided to 34 individuals. Training included classroom sessions, mock interviews, and field practice. Data collection took place between January 2 and June 15, 2023, by 30 field teams. Each team included a supervisor, interviewers, a biomarker technician, and drivers. Data was transmitted to the Department of Statistics (DoS) using a secure platform (SynCloud). Electronic data files were securely stored and processed at the DoS central office. Data editing involved resolving inconsistencies and coding responses using CSPro. Quality checks and feedback were ongoing during fieldwork, with final data processing completed by September 2023 [21].

### Outcome of interest

The primary outcomes were cervical and breast cancer screening. Participants were asked three questions: (1) “*Has a doctor or healthcare provider ever tested you for cervical cancer*?”, (2) “*Has a doctor or healthcare provider examined your breasts to check for breast cancer*?”* and* (3) “*Have you ever had a mammogram*?” [21]. Responses were recorded as “yes,” “no,” or “don’t know.” The cervical cancer screening outcome was based on the first question and coded as binary indicators (1 = yes, 0 = no) [21]. The breast cancer screening outcome was derived from responses to questions 2 and 3. It was coded as (1) 1 (yes) if the response to either question 2 or question 3 was “yes,”; (2) 0 (no) only if both responses were “no,” and (3) missing if responses to both questions were “don’t know” [21]. Participants who responded “don’t know” were excluded from the analysis. Both variables were coded as binary indicators (1 = yes, 0 = no).

### Explanatory variables

Based on the literature review and biological and social plausibility [22–26], the following explanatory variables were included in the analysis: The independent variables included age group, wealth index, education, parity group, healthcare visit within the previous 12 months of the survey, distance to health facility, transport to health facility, health insurance, work status, contraceptive use, place of residence, sexually transmitted infection (STI) status, frequency of reading newspapers, frequency of listening to the radio, and frequency of watching television. The detailed categorization of the variables is mentioned in Table 1. The wealth index was derived using information collected in the JPFHS 2023 on asset ownership, such as housing construction materials, water sources, sanitation facilities, electricity usage, and access to health services. Principal component analysis was performed to calculate the wealth index, which was then categorized into quintiles [21, 27–29].

**Table 1:** List of study variables

Study variables	Description and categories
Outcome variables	
1. Breast cancer screening	Ever underwent breast cancer screening (0 = No; 1 = Yes)
2. Cervical cancer screening	Ever underwent cervical cancer screening (0 = No; 1 = Yes)
Explanatory variables	
1. Age group	Age group of the participants in years (0 = 15–24 years; 1 = 24–34 years; 2 = 35–49 years)
2. Wealth Index	Household wealth quintile (0 = poorest; 1 = poorer; 2 = middle; 3 = richer; 4 = richest)
3. Education	Education level of the participants (0 = no formal education; 1 = primary; 2 = secondary or above)
4. Parity	The number of pregnancies reaching viable gestational age (including live births and stillbirths) (0 = 0 child; 1 = 1 child; 2 = 2 children; 3 = ≥ 3 children)
5. Healthcare visit	Healthcare visit within previous 12 months of the survey (0 = No; 1 = Yes)
6. Distance to health facility	Distance to the nearest health facility measured in time (0 = ≤ 10 min; 1 = 11–20 min; 2 = > 20 min)
7. Transport to health facility	Medium of transportation to the health facility (0 = Motorized; 1 = Non-motorized/others)
8. Health insurance	Health insurance status of the participants (0 = No; 1 = Yes)
9. Work status	Whether the participant was working during the time of the survey (0 = Not working; 1 = Working)
10. Contraceptive use	Contraceptive use status of the participants (0 = No; 1 = Yes)
11. Place of residence	Type of the cluster (0 = Urban; 1 = Rural)
12. Sexually transmitted infection	Sexually transmitted infection status of the participants (0 = No/Don’t know; 1 = Yes)
13. Frequency of reading newspapers	Participants’ frequency of reading newspapers (0 = Not at all; 1 = Less than once a week; 2 = At least once a week)
14. Frequency of listening to radio	Participants’ frequency of listening to radio (0 = Not at all; 1 = Less than once a week; 2 = At least once
15. Frequency of watching television	Participants’ frequency of watching television (0 = Not at all; 1 = Less than once a week; 2 = At least once

### Statistical analysis

Data analysis was performed using Stata version 18.0, and the study adhered to the Strengthening the Reporting of Observational Studies in Epidemiology (STROBE) guidelines. A complete case analysis was conducted, as the proportion of missing data was minimal (less than 1%). Sampling weights were applied during the study to account for the complex survey design. A descriptive analysis presented results as unweighted counts and weighted proportions. The prevalence rates for breast and cervical cancer screenings were estimated, and chi-square tests were used to assess differences in screening prevalence across selected independent variables. Multivariable logistic regression models were employed to identify factors associated with each screening type, with both crude odds ratios (COR) and adjusted odds ratios (AOR) reported alongside 95% confidence intervals (CI). Statistical significance was defined as *p* < 0.05.

### Ethical consideration

The JPFHS 2023 protocol received approval from the ICF Institutional Review Board (IRB). Participants provided informed consent at the time of the survey [21], including consent for the use of anonymized data in future research. The publicly available DHS dataset is fully anonymized, with no personal identifiers or precise geographic information, in accordance with IRB-approved confidentiality protocols [21]. For this secondary analysis, anonymized data were obtained from the DHS Program following the approval of a research proposal. Since the analysis utilized de-identified data, no additional ethical approval was necessary.

## Findings

### Background characteristics of participants

The final analytical sample comprised 12,547 participants (Fig. 1).

Table 2 presents the sociodemographic characteristics and screening status for cervical and breast cancer across key covariates. Participants were predominantly aged 35–49 years (59.5%), with 31.9% aged 25–34 years and 8.6% aged 15–24 years. Socioeconomic variation was evident, with 40.4% belonging to the poorest or poorer wealth quintiles, and over half (57.0%) having attained secondary education, while 34.6% had higher education. A majority (66.4%) had accessed a healthcare facility within the 12 months preceding the survey, and 55.5% reported the current use of contraceptives. Most participants resided in urban areas (91.1%), and nearly one-third (31.0%) lacked health insurance coverage.

**Table 2:** Background characteristics of participants and prevalence of cervical cancer and breast cancer screening status by covariates, Jordan Population and Family Health Survey 2023 (*N* = 12,547)

**Variable**	**Overall sample,** ***n* (%)**	**Prevalence of breast cancer screening (%)**	*p* value*	**Prevalence of cervical cancer screening (%)**	*p* value**
Age group			< 0.001		< 0.001
15–24	1204 (8.6)	4.2		3.4	
25–34	4205 (31.9)	9.1		10.8	
35–49	7138 (59.4)	20.0		20.9	
Wealth index			< 0.001		< 0.001
Poorest	3780 (19.5)	9.9		8.0	
Poorer	2787 (20.9)	10.6		10.7	
Middle	2536 (21.4)	15.4		16.4	
Richer	2175 (19.6)	18.0		21.5	
Richest	1269 (18.5)	22.7		25.2	
Education			< 0.05		< 0.001
No education or primary	1476 (8.3)	10.5		7.8	
Secondary	7259 (57.0)	15.1		15.1	
Higher	3812 (34.6)	16.5		20.0	
Parity			< 0.001		< 0.001
0 child	1002 (7.8)	7.6		8.0	
1 child	1227 (10.1)	8.6		10.8	
2 children	1953 (17.0)	12.1		16.3	
≥ 3 children	8365 (65.0)	17.9		18.0	
Healthcare visit			> 0.05		> 0.05
No	4034 (33.6)	13.5		15.4	
Yes	8513 (66.4)	16.0		16.6	
Distance to health facility			> 0.05		< 0.01
≤ 10 min	6829 (54.9)	16.2		17.7	
11–20 min	3948 (31.3)	14.3		14.9	
> 20 min	1770 (13.8)	13.0		13.1	
Transport to health facility			> 0.05		> 0.05
Motorized	7311 (67.1)	15.4		16.8	
Non-motorized/Others	5236 (32.9)	14.8		14.9	
Health insurance			> 0.05		> 0.05
No	2871 (31.0)	14.0		16.7	
Yes	9676 (69.0)	15.7		16.0	
Work status			< 0.05		< 0.01
Not working	10,950 (86.4)	14.7		15.5	
Working	1597 (13.6)	18.4		20.8	
Contraceptive use			< 0.05		< 0.05
Not using	5847 (44.5)	13.6		14.9	
Using	6700 (55.5)	16.4		17.2	
Place of residence			< 0.05		< 0.01
Urban	10,399 (91.1)	15.5		16.7	
Rural	2148 (8.9)	11.5		11.0	
STI			< 0.001		< 0.001
No/Don't know	11,502 (93.1)	14.4		15.5	
Yes	1045 (6.9)	25.2		25.1	
Frequency of reading newspapers			> 0.05		< 0.05
Not at all	9850 (76.2)	14.5		15.4	
Less than once a week	1126 (10.0)	17.8		20.2	
At least once a week	1571 (13.8)	17.1		17.8	
Frequency of listening to radio			< 0.001		< 0.001
Not at all	9187 (70.6)	13.7		14.9	
Less than once a week	1494 (13.3)	20.0		22.3	
At least once a week	1866 (16.1)	17.8		16.7	
Frequency of watching television			> 0.05		> 0.05
Not at all	2054 (16.0)	13.1		13.7	
Less than once a week	2432 (18.3)	16.8		17.1	
At least once a week	8061 (65.7)	15.2		16.5	

### Prevalence of breast and cervical cancer screening

The overall prevalence of breast and cervical cancer screening was 15.2% and 16.2%, respectively. Screening uptake for both cancers increased significantly with age (*p* < 0.001), education level, and wealth status. The highest screening rates were observed among participants in the richest wealth quintile (breast cancer: 22.7%, *p* < 0.001; cervical cancer: 25.2%, *p* < 0.001) and those with higher education (breast cancer: 16.5%, *p* < 0.05; cervical cancer: 20.0%, *p* < 0.001). The screening prevalence increased progressively from nulliparous women to those with three or more children (*p* < 0.001 for both outcomes).

Additional correlates of higher screening uptake included employment at the time of the survey (breast cancer: 18.4% vs. 14.7%, *p* < 0.05; cervical cancer: 20.8% vs. 15.5%, *p* < 0.001), contraceptive use (breast cancer: 16.4% vs. 13.6%; cervical cancer: 17.2% vs. 14.9%; *p* < 0.05 for both), and urban residence (breast cancer: 15.5% vs. 11.5%, *p* < 0.05; cervical cancer: 16.7% vs. 11.0%, *p* < 0.01). Participants who reported a history of sexually transmitted infections had substantially higher screening rates compared to those who did not or were unsure (breast cancer: 25.2% vs. 14.4%, *p* < 0.001; cervical cancer: 25.1% vs. 15.5%, *p* < 0.001) (Table 2).

### Factors associated with breast cancer screening

In the final multivariable logistic regression model, breast cancer screening in Jordan was significantly associated with age, wealth status, parity, healthcare visits, STI history, and frequency of listening to the radio (Table 3). The odds of screening increased significantly with age: women aged 25–34 had 1.8 times higher odds (95% CI: 1.1–2.7), and those aged 35–49 had 4.0 times higher odds (95% CI: 2.6–6.0) compared to women aged 15–24. Screening likelihood also rose with increasing wealth: compared to the poorest group, women in the middle (AOR: 1.6; 95% CI: 1.2–1.9), richer (AOR: 1.7; 95% CI: 1.3–2.1), and richest (AOR: 2.1; 95% CI: 1.6–2.8) quintiles had significantly higher odds of screening.

**Table 3:** Logistic regression analysis results of factors associated with breast cancer screening in Jordan, Jordan Population and Family Health Survey 2023

Variable	COR (95% CI)	AOR (95% CI)
Age group		
15–24	Ref	Ref
25–34	2.3*** (1.5–3.6)	1.8* (1.1–2.7)
35–49	5.8*** (3.9–8.7)	4.0*** (2.6–6.0)
Wealth index		
Poorest	Ref	Ref
Poorer	1.1 (0.9–1.4)	1.0 (0.8–1.2)
Middle	1.7*** (1.3–2.1)	1.6** (1.2–1.9)
Richer	2.0*** (1.6–2.6)	1.7*** (1.3–2.1)
Richest	2.7*** (2.1–3.5)	2.1*** (1.6–2.8)
Education		
No education or primary	Ref	Ref
Secondary	1.5* (1.1–2.1)	1.2 (0.9–1.9)
Higher	1.7** (1.2–2.4)	1.1 (0.7–1.9)
Parity		
0 child	Ref	Ref
1 child	1.1 (0.7–1.9)	1.0 (0.6–1.5)
2 children	1.7* (1.1–2.5)	1.3 (0.8–1.8)
≥ 3 children	2.7*** (1.8–3.9)	1.6* (1.1–2.1)
Healthcare visit		
No	Ref	Ref
Yes	1.2 (1.–1.5)	1.3** (1.1–1.6)
Distance to health facility		
≤ 10 min	Ref	Ref
11–20 min	0.9 (0.7–1.1)	0.9 (0.7–1.2)
> 20 min	0.8* (0.6–1.0)	0.9 (0.7–1.2)
Transport to health facility		
Motorized	Ref	Ref
Non-motorized/Others	1.0 (0.8–1.1)	1.0 (0.9–1.1)
Health insurance		
No	Ref	Ref
Yes	1.1 (1.0–1.4)	1.1 (0.9–1.3)
Work status		
Not working	Ref	Ref
Working/Other	1.3* (1.0–1.6)	1.1 (0.8–1.4)
Contraceptive use		
Not using	Ref	Ref
Using	1.2* (1.0–1.5)	1.0 (0.8–1.3)
Place of residence		
Urban	Ref	Ref
Rural	0.7* (0.5–0.9)	0.8 (0.6–1.1)
STI		
No/Don’t know	Ref	Ref
Yes	2.0*** (1.5–2.6)	2.1*** (1.6–3.1)
Frequency of reading newspapers		
Not at all	Ref	Ref
Less than once a week	1.3* (1.0–1.6)	0.9 (0.7–1.1)
At least once a week	1.2 (0.9–1.6)	1.0 (0.8–1.6)
Frequency of listening to radio		
Not at all	Ref	Ref
Less than once a week	1.6*** (1.3–2.0)	1.4** (1.1–1.6)
At least once a week	1.4** (1.1–1.7)	1.2 (0.9–1.4)
Frequency of watching television		
Not at all	Ref	Ref
Less than once a week	1.3* (1.1–1.7)	1.1 (0.9–1.5)
At least once a week	1.2 (1.0–1.5)	1.0 (0.8–1.2)

*CI* confidence interval, *COR* crude odds ratio, *AOR* adjusted odds ratio.*

*p < 0.05, **p < 0.01, ***p < 0.001.

Women with three or more children had higher odds of screening compared to those without children (AOR: 1.6; 95% CI: 1.1–2.1). Healthcare facility visits were associated with increased screening (AOR: 1.3; 95% CI: 1.1–1.6). A history of STIs was strongly associated with screening (AOR: 2.1; 95% CI: 1.6–3.1). Regarding media exposure, women who listened to the radio less than once a week had significantly higher odds of screening (AOR: 1.4; 95% CI: 1.1–1.6) than non-listeners. No significant associations were observed for education, transportation mode, health insurance coverage, contraceptive use, or other forms of media exposure (newspaper or television) in the adjusted model.

### Factors associated with cervical cancer screening

In the adjusted analysis, cervical cancer screening in Jordan was significantly associated with age, education, wealth, parity, place of residence, STIs, and frequency of radio listening (Table 4). Like breast cancer screening, the odds of cervical cancer screening increased with increasing age and wealth index. Compared to women aged 15–24, those aged 25–34 years (AOR: 2.6; 95% CI: 1.6–4.3) and 35–49 years (AOR: 5.5; 95% CI: 3.3–9.2) had significantly higher odds of screening. Women in the middle (AOR: 1.9; 95% CI: 1.5–2.6), richer (AOR: 2.4; 95% CI: 1.8–3.2), and richest (AOR: 2.6; 95% CI: 1.9–3.5) groups were more likely to be screened compared to the poorest quintile. Education was also independently associated with screening, with women having secondary (AOR: 1.6; 95% CI: 1.1–2.2) and higher education (AOR: 1.6; 95% CI: 1.2–2.3) showing increased odds compared to those with no education or educated up to primary education. Greater parity was associated with increased screening; women with two children (AOR: 1.7; 95% CI: 1.2–2.6) or three or more (AOR: 1.6; 95% CI: 1.1–2.3) had higher odds than nulliparous women. Participants reporting a history of STIs had significantly higher odds of screening (AOR: 1.8; 95% CI: 1.4–2.4; *p* < 0.001), and those residing in rural areas had lower odds compared to those living in the urban areas (AOR: 0.7; 95% CI: 0.6–1.0; *p* < 0.05). Listening to the radio less than once a week was also positively associated with screening compared to listening not at all (AOR: 1.3; 95% CI: 1.0–1.7). Other factors, including healthcare visits, health insurance, and contraceptive use, were not significantly associated with cervical cancer screening in the adjusted model.

**Table 4:** Logistic regression analysis results of factors associated with cervical cancer screening in Jordan, Jordan Population and Family Health Survey 2023

Variable	COR (95% CI)	AOR (95% CI)
Age group		
15–24	Ref	Ref
25–34	3.4*** (2.0–5.6)	2.6*** (1.6–4.3)
35–49	7.4*** (4.6–12.0)	5.5*** (3.3–9.2)
Wealth index		
Poorest	Ref	Ref
Poorer	1.4* (1.1–1.8)	1.2 (1.0–1.6)
Middle	2.3*** (1.7–3.0)	1.9*** (1.5–2.6)
Richer	3.2*** (2.4–4.1)	2.4*** (1.8–3.2)
Richest	3.9*** (2.9–5.2)	2.6 *** (1.9–3.5)
Education		
No education or primary	Ref	Ref
Secondary	2.1*** (1.5–2.9)	1.6*** (1.1–2.2)
Higher	3.0*** (2.1–4.1)	1.6*** (1.2–2.3)
Parity		
0 child	Ref	Ref
1 child	1.4 (0.9–2.2)	1.3 (0.8–2.0)
2 children	2.2*** (1.5–3.3)	1.7*** (1.2–2.6)
≥ 3 children	2.5*** (1.8–3.5)	1.6*** (1.1–2.3)
Healthcare visit		
No	Ref	Ref
Yes	1.1 (0.9–1.4)	1.2 (1.0–1.5)
Distance to health facility		
≤ 10 min	Ref	Ref
11–20 min	0.8* (0.7–1.0)	0.9 (0.7–1.1)
> 20 min	0.7** (0.5–0.9)	0.9 (0.7–1.2)
Transport to health facility		
Motorized	Ref	Ref
Non-motorized/Others	0.9 (0.7–1.0)	1.0 (0.8–1.2)
Health insurance		
No	Ref	Ref
Yes	0.9 (0.8–1.2)	0.9 (0.7–1.1)
Work status		
Not working	Ref	Ref
Working/Other	1.4*** (1.2–1.7)	1.1 (0.9–1.3)
Contraceptive use		
Not using	Ref	Ref
Using	1.2* (1.0–1.4)	1.0 (0.8–1.1)
Place of residence		
Urban	Ref	Ref
Rural	0.6** (0.5–0.8)	0.7*** (0.6–1.0)
STI		
No/Don’t Know	Ref	Ref
Yes	1.8*** (1.4–2.3)	1.8*** (1.4–2.4)
Frequency of reading newspapers		
Not at all	Ref	Ref
Less than once a week	1.4** (1.1–1.8)	1.0 (0.8–1.3)
At least once a week	1.2 (0.9–1.5)	1.1 (0.8–1.4)
Frequency of listening to radio		
Not at all	Ref	Ref
Less than once a week	1.6*** (1.3–2.0)	1.3*** (1.0–1.7)
At least once a week	1.1 (0.9–1.5)	0.9 (0.7–1.2)
Frequency of watching television		
Not at all	Ref	Ref
Less than once a week	1.3 (1.0–1.7)	1.1 (0.8–1.5)
At least once a week	1.2 (1.0–1.6)	1.1 (0.8–1.4)

*CI* confidence interval, *COR* crude odds ratio, *AOR* adjusted odds ratio

*p < 0.05, **p < 0.01, ***p < 0.001

## Discussion

This study aimed to determine the prevalence and factors associated with breast and cervical cancer screening among ever-married women aged 15–49 years in Jordan using nationally representative data from the JPFHS 2023. We found that 15.2% of women had undergone breast cancer screening, and 16.2% had received cervical cancer screening. Breast and cervical cancer screening in Jordan were both significantly associated with age, wealth status, parity, history of STIs, and frequency of listening to the radio. Additionally, breast cancer screening was associated with recent healthcare visits, while cervical cancer screening was associated with education and place of residence.

The 2017–2018 version of the JPFHS found that 14% of ever-married women of reproductive age had undergone a breast examination by a health specialist, and 9% had ever had a mammogram [17]. Overall, the prevalence of breast cancer screening remained unchanged over the 5 years, indicating a continued need for programmatic efforts to improve screening uptake. A recent study conducted among 650 Jordanian women aged 20 years and above in an urban setting found that only 10.5% (*n* = 68) reported routinely receiving clinical breast exams. Regarding mammography, approximately one-fifth of participants (20.0%, *n* = 130) had undergone the procedure at least once in their lifetime; however, only 11.1% (*n* = 72) reported undergoing mammograms regularly. These differences may be attributed to variations in sample size, study methodology, and the target population [6]. Similarly, the prevalence of cervical cancer screening was 15.8% in 2017–2018, suggesting only a 0.4% increase over the 5 years [7].

Findings from a recent review of breast cancer screening practices in the Middle East revealed substantial variability in screening uptake across countries. Mammography use ranged from 1.6% in Yemen to 69.4% in Lebanon, with rates in Saudi Arabia varying between 12.8 and 40%. Clinical breast examinations were practiced by 18.9% to 21.9% of women in Saudi Arabia and Yemen. In contrast, breast self-examination rates showed wider variation, ranging from 26% in Iraq to 57.5% in Oman for forever practicing and 17.4% (Palestine) to 27.9% (Saudi Arabia) for routine practice. Despite relatively high awareness, screening uptake remains low in many contexts, highlighting persistent structural, cultural, and informational barriers. These patterns are consistent with the low screening prevalence observed in Jordan and reinforce the need for targeted, context-specific interventions to improve screening behaviors across the region [30].

Similarly, a recent meta-analysis involving 55 studies and over 204,000 Arab women reported an overall cervical cancer screening uptake rate of 18.2% (95% CI: 13.9–23.6%) across Arab countries [31]. However, substantial disparities were observed between countries. Bahrain had the highest reported prevalence at 44.1%, followed by Lebanon at 42.2%, while Somalia had the lowest at 8.9% [31]. In Jordan, the pooled prevalence was 26.3% (95% CI: 20.5–32.2%), which is relatively higher than the regional average [31].

Consistent with existing literature, our analysis revealed that the likelihood of screening for both cancers increased significantly with age [32–37]. Women aged 25–34 and 35–49 years had greater odds of being screened than those aged 15–24. This pattern may reflect increased health awareness and perceived vulnerability among older women and their more frequent interaction with healthcare services [37]. Additionally, the higher susceptibility to breast and cervical cancer with increasing age likely contributes to their increased engagement in screening practices [37].

Healthcare utilization within the previous 12 months was positively associated with breast cancer screening, indicating that routine contact with the health system may provide opportunities for promoting early detection. Although health insurance coverage was not a significant predictor in our adjusted models for breast cancer screening, it was positively associated with cervical cancer screening in the bivariate analysis, aligning with findings from other settings that link financial access to preventive care utilization [24].

Wealth index was found to be associated with both breast and cervical cancer screening. This is consistent with prior findings highlighting the role of socioeconomic status in accessing preventive health services [38–40]. Educational attainment showed no significant association with breast cancer screening in the adjusted model. Still, it was independently associated with cervical cancer screening, with higher odds among women with secondary and higher education. These results underscore the importance of educational interventions in improving awareness and health-seeking behavior, particularly for cervical cancer screening, where knowledge gaps may limit participation [41].

Urban residence was associated with higher screening rates for both breast and cervical cancers, suggesting geographical disparities in access to care. Women residing in rural areas had significantly lower odds of cervical cancer screening, reflecting persistent inequities in healthcare infrastructure, outreach, and service delivery. These findings emphasize the need for targeted interventions in rural areas to improve access to screening services [42].

The history of STIs was associated with increased rates of both breast and cervical cancer screening. This likely reflects greater medical engagement among women with an STI history and provider-initiated opportunities to offer cancer screening during STI-related consultations [43].

Exposure to mass media, particularly radio, also significantly associated with increased screening uptake. This suggests that targeted public health messaging through radio could be an effective strategy for raising awareness, especially in populations with limited access to other forms of media [44, 45].

To improve cancer screening uptake in Jordan, efforts should focus on integrating screening into routine healthcare services, enhancing outreach in rural and underserved areas, and expanding education and awareness campaigns. Reducing financial and informational barriers, especially for lower-income and less-educated women will help to promote equitable access.

Previous reviews have identified several common barriers to breast and cervical cancer screening in Arab countries, including Jordan [30, 31]. These include a lack of knowledge and awareness about the importance and availability of screening services, as well as a low perceived risk, particularly among asymptomatic women. Fear of diagnosis, embarrassment, and discomfort with the procedures, especially in settings where access to female healthcare providers is limited, also emerged as significant deterrents [30, 31]. Additionally, financial constraints, cultural sensitivities, and social stigma were frequently reported as barriers to both mammography and Pap smear uptake [30, 31]. Both studies found that women who had undergone screening exhibited higher perceived benefit scores and lower perceived barriers, underscoring the critical role of health literacy, community engagement, and culturally tailored interventions in improving screening behaviors [30, 31]. These shared challenges reinforce the need for integrated, multi-level strategies to increase cancer screening uptake among women in Jordan and the broader Arab region [30, 31].

The JBCP, led by the King Hussein Cancer Foundation and Center in partnership with the Ministry of Health, provides national guidelines for breast cancer screening. Women aged 25–39 are advised to perform monthly self-breast exams (SBE) and undergo annual clinical breast exams (CBE), while those aged 40 and above are also recommended to receive yearly mammograms [16, 46]. Opportunistic cervical cancer screening is conducted across public and private healthcare sectors using cytology-based methods. Additionally, Jordan implements nationwide outreach initiatives to encourage women to participate in screening programs [47]. Despite these efforts, screening uptake remains low and appears to be declining, primarily due to financial barriers and limited insurance coverage [6].

### Limitations of the study

This study offers valuable insights into breast and cervical cancer screening behaviors among ever-married women in Jordan using nationally representative data. However, several limitations should be noted. The use of secondary data limited the inclusion of important variables such as personal health beliefs, cultural norms, and healthcare provider influences. The cross-sectional design prevents causal interpretation of associations. Self-reported screening behaviors may be affected by recall or social desirability bias. The study population was restricted to ever-married women aged 15–49 years, excluding never-married and older women, which limits generalizability. In addition, the absence of provider- and facility-level data constrains the assessment of supply-side barriers. Geographic disparities beyond rural–urban residence were not fully explored. Future studies should adopt longitudinal and mixed-methods approaches to address these gaps.

### Future directions

The present study highlights critical sociodemographic disparities in breast and cervical cancer screening participation among Jordanian women, underscoring the need for deeper inquiry and targeted interventions. Building on these findings, several avenues for future research are recommended. First, qualitative studies are essential to explore the nuanced social, cultural, and psychological barriers that limit screening uptake, particularly among young, rural, and less educated women. Such research could uncover factors not captured in quantitative surveys, such as perceptions of cancer risk, stigma surrounding pelvic examinations, and trust in healthcare providers. Second, longitudinal studies should be conducted to better understand causal pathways between sociodemographic variables and screening behaviors over time. These would help clarify how evolving life circumstances such as income changes, education, or healthcare access affect a woman’s likelihood of undergoing cancer screening. Third, research evaluating the implementation and reach of Jordan’s national screening programs is necessary to identify systemic and logistical barriers at the provider and facility levels. Investigating availability, accessibility, referral processes, and provider training could provide actionable insights to strengthen service delivery. Fourth, intervention studies that assess the effectiveness of media-based outreach (e.g., radio, television, or digital platforms) and mobile health (mHealth) strategies, such as SMS reminders or app-based education should be prioritized. Given the positive association between radio exposure and screening participation observed in this study, such interventions may hold promise for enhancing awareness and engagement. Finally, future research should broaden the population scope beyond ever-married women aged 15 to 49 to include never-married and older women, who may have differing risk profiles and access barriers. Additionally, intersectional analyses that examine how overlapping factors such as poverty, rurality, and low education compound disadvantage would contribute to more equitable policy formulation. By addressing these research gaps, future studies can help inform more inclusive and effective cancer screening strategies that reduce disparities and improve early detection outcomes in Jordan and similar low- and middle-income settings.

### Policy recommendation

To improve breast and cervical cancer screening rates in Jordan, policy interventions must directly address the underlying sociodemographic disparities that limit access and participation. Targeted efforts should focus on younger women, individuals with lower levels of education, and those living in rural or economically disadvantaged areas. Expanding community-based awareness campaigns using accessible and culturally relevant platforms such as radio, social media, and local gatherings can effectively reach women with limited access to healthcare services. Integrating cancer screening into routine health checkups and primary care visits, along with provider-led encouragement and potential incentives, can significantly improve screening uptake. Educational initiatives that promote knowledge about cancer prevention and the importance of early detection should be intensified, especially within underserved communities. Finally, equitable access to national screening programs must be ensured through active promotion and strategic allocation of resources, closing current gaps and improving early diagnosis and health outcomes across the population.

## Conclusion

This study highlights the essential sociodemographic and contextual factors affecting breast and cervical cancer screening participation among women in Jordan. Crucial determinants including age, socioeconomic position, parity, history of sexually transmitted infections, and exposure to radiation were significantly correlated with both forms of screening. Moreover, breast cancer screening had a positive correlation with recent healthcare consumption, but cervical cancer screening was affected by educational attainment and geographic location. To enhance screening coverage, focused initiatives should target younger women, those from lower socioeconomic backgrounds, and nulliparous individuals. Initiatives to improve cervical cancer screening should prioritize women with less education and those residing in remote regions. These findings provide critical insights for policymakers and healthcare providers to develop and implement evidence-based, equity-oriented strategies that address existing disparities and improve the accessibility and effectiveness of cancer screening services within the Jordanian healthcare system.

## Data Availability

Data are available from the Demographic and Health Surveys (DHS) Program. Following the proper application procedure, the data can be accessed at: https://dhsprogram.com
